# Nanobody-Based Immunoassays for the Detection of Food Hazards—A Review

**DOI:** 10.3390/bios15030183

**Published:** 2025-03-13

**Authors:** Wenkai Li, Zhihao Xu, Qiyi He, Junkang Pan, Yijia Zhang, El-Sayed A. El-Sheikh, Bruce D. Hammock, Dongyang Li

**Affiliations:** 1 College of Biosystems Engineering and Food Science, Zhejiang University, Hangzhou 310058, China; 12313055@zju.edu.cn (W.L.); zhihaoxu@zju.edu.cn (Z.X.); chesto36@163.com (Q.H.); kang36576@163.com (J.P.); zyj1016779121@163.com (Y.Z.); 2 Key Laboratory of Intelligent Equipment and Robotics for Agriculture of Zhejiang Province, Hangzhou 310058, China; 3 Faculty of Agriculture, Zagazig University, Zagazig 44511, Sharkia, Egypt; eaelsheikh@agri.zu.edu.eg; 4 Department of Entomology and Nematology and UCD Comprehensive Cancer Center, University of California Davis, Davis, CA 95616, USA

**Keywords:** nanobody, immunoassay, food hazards, food safety

## Abstract

Food safety remains a significant global challenge that affects human health. Various hazards, including microbiological and chemical threats, can compromise food safety throughout the supply chain. To address food safety issues and ensure public health, it is necessary to adopt rapid, accurate, and highly specific detection methods. Immunoassays are considered to be an effective method for the detection of highly sensitive biochemical indicators and provide an efficient platform for the identification of food hazards. In immunoassays, antibodies function as the primary recognition elements. Nanobodies have significant potential as valuable biomolecules in diagnostic applications. Their distinctive physicochemical and structural characteristics make them excellent candidates for the development of reliable diagnostic assays, and as promising alternatives to monoclonal and polyclonal antibodies. Herein, we summarize a comprehensive overview of the status and prospects of nanobody-based immunoassays in ensuring food safety. First, we begin with a historical perspective on the development of nanobodies and their unique characteristics. Subsequently, we explore the definitions and boundaries of immunoassays and immunosensors, before discussing the potential applications of nanobody-based immunoassays in food safety testing that have emerged over the past five years, and follow the different immunoassays, highlighting their advantages over traditional detection methods. Finally, the directions and challenges of nanobody-based immunoassays in food safety are discussed. Due to their remarkable sensitivity, specificity and versatility, nanobody-based immunoassays hold great promise in revolutionizing food safety testing and ensuring public health and well-being.

## 1. Introduction

The globalization of food supply chains and the increasing demand for food micro-processing have elevated food safety to a critical global public health issue [1]. Foodborne illnesses resulting from food contamination can lead to considerable morbidity, mortality, and significant economic costs, creating major public health, economic, and social challenges worldwide [2]. In 2015, the World Health Organization (WHO) reported that approximately one in ten individuals fall ill because of food contamination by microbial or chemical agents. Foodborne diseases accounted for 600 million cases, 420,000 deaths (40% among children under five), and a loss of 33 million healthy life years worldwide, with nearly 29% of these cases attributed to the transmission of contaminated food (582 million cases) [3]. Currently, over 200 foodborne illnesses are transmitted through the consumption of foods contaminated with biohazardous and chemical agents [4]. Food safety is significantly compromised by the presence of harmful substances, with chemical and biological hazards being the primary causes of food poisoning and foodborne illnesses. Chemical hazards encompass environmental contaminants found in soil and water as well as residues from pesticides, fisheries, and veterinary medications used in animal husbandry. Biological hazards mainly involve various forms of contamination caused by biotoxins and foodborne pathogens [5]. Given the dangers posed by food hazards and their global impact, there is a pressing need to develop rapid, reliable, sensitive, and user-friendly detection technologies. Such advancements are crucial for safeguarding public health and well-being, making this area a focal point of current research efforts [6,7,8].

Currently, there are a number of well-established instruments that can accurately and quantitatively detect contaminants in food, including thin-layer chromatography (TLC) [9], gas chromatography–mass spectrometry (GC-MS) [10], liquid–liquid-mass spectrometry (LC-MS) [11], and high-performance liquid chromatography–mass spectrometry (HPLC-MS) [12]. They are widely used for the detection of food hazards with very high sensitivity and good selectivity. Nonetheless, the use of instrumental methods requires expensive equipment and the expertise of trained personnel to handle the samples, which entails considerable expense and a great deal of time. Traditional methods, including culturing in combination with biochemical identification, are usually confined to the laboratory environment and are not suitable for field testing. Therefore, there is a growing demand for the development of simple, rapid, low-cost, and portable analytical methods for food safety testing.

Immunoassays are characterized by fast analytical speed, high sensitivity, good selectivity, and versatility [13]. They are now widely used in the detection of food hazards such as pathogenic bacteria [14], allergens [15], and pesticide residues [16]. Immunoassays operate on the principle of antigen–antibody binding to ascertain the presence or concentration of antigens by quantifying the signal resulting from the interaction between antibodies and antigens. In immunoassays, conventional antibodies serve as biorecognition elements, primarily consisting of polyclonal antibodies (pAbs) derived from immunized mouse or rabbit sera, or monoclonal antibodies (mAbs) produced using hybridoma technology [16]. However, in contemporary immunoassay applications, acquiring antibodies with high affinity and specificity is crucial to ensure their long shelf life and capacity to endure extreme environmental conditions over prolonged periods [17]; mAbs entail lengthy preparation cycles, high costs, and inadequate stability, whereas pAbs exhibit limitations such as low specificity and susceptibility to non-specific binding. Furthermore, both pAbs and mAbs often lack sufficient validation, which represents a significant limitation of immunoassays [18]. To achieve sensitive detection in food safety, the recent advancement in antibody engineering technology has facilitated the production of a variety of antibodies, including nanobodies with variable structural domains derived from naturally occurring pure heavy-chain antibodies in camelids [19] (such as llamas, alpacas, or camels) and sharks [20], which are an exciting frontier in recombinant antibody research. In contrast to conventional mAbs and pAbs, which comprise two heavy chains and two light chains containing variable structural domains (VH and VL) that participate in forming the complementary sites of the antibody, camelid HcAbs consist solely of a heavy chain. Its antigen-binding site is exclusively formed by heavy-chain variable domains (VHH) and is commonly referred to as a nanobody (Nb) because of its remarkably low molecular weight, typically ranging from 12 to 14 kDa. Compared with traditional mAbs and pAbs, Nbs offer advantages such as simple preparation, low cost, good stability, low immunogenicity, and ease of modification and expression. Therefore, Nbs are promising candidates as recognition proxies to enhance detection sensitivity and stability in immunoassays [21].

**Figure 1:**
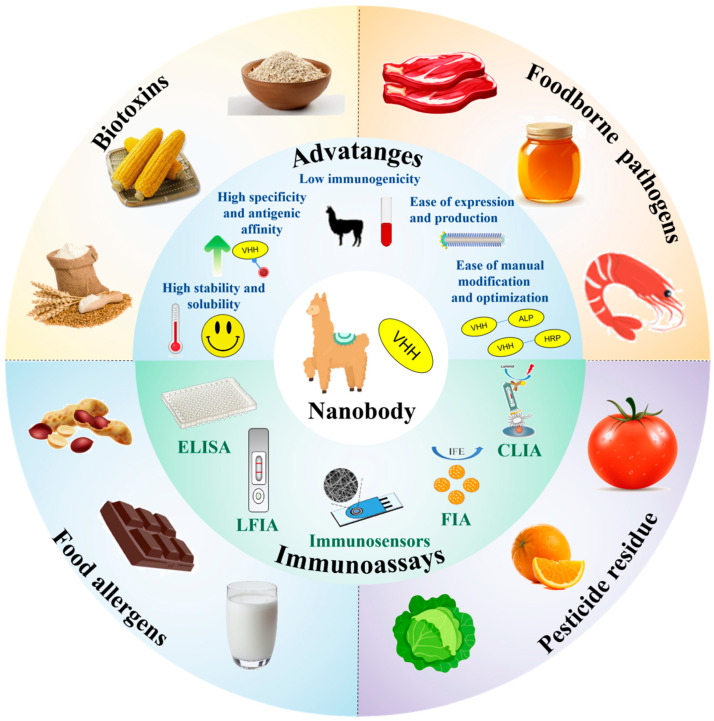
Schematic diagram of immunoassays based on Nbs for food safety detection, including the characteristics of Nbs and signal output methods for immunoassay strategies, including enzyme-linked immunosorbent assay (ELISA), lateral flow immunoassay (LFIA), immunosensors, and other methods.

In recent years, an increasing number of Nb-based immunoassay techniques have been developed and applied for the detection of food hazards. Reviews on the use of Nbs in analytical applications have been published in recent years [22,23]. Recently, research on nanobody-based immunosensors for the analysis of food hazards has been reviewed elsewhere [24]. In this review, we provide a summary of nanobody-based immunoassays for detecting food hazards. A clear distinction between immunosensors and immunoassays is rarely discussed in the literature, with the terms frequently used interchangeably. The development of Nb-based immunoassay techniques also requires more in-depth elaboration from the concept to the application of Nbs. In this paper, we review the recent applications of Nb-based immunoassay technology for food hazard detection. First, we describe the association and difference between immunoassay methods and immunosensors so that readers can have a clearer perception. Secondly, we review the history of Nbs and outline their functional properties. Subsequently, we highlight recent advances in Nb-based immunoassays for food safety testing over the past five years, focusing on biotoxins, pathogens, allergens, and pesticide residues, with a hierarchical presentation according to different immunoassay methods (Figure 1). Finally, we discuss the future development and challenges associated with Nbs, with the hope that this paper will provide further research on Nbs in immunoassays.

## 2. Overview of Nb

### 2.1. Brief History of Nb

The core function of antibodies, which are integral to the adaptive immune system as proteins, is to specifically recognize and bind to antigenic epitopes on the surfaces of foreign molecules. This interaction facilitates antigen neutralization and removal while maintaining immune homeostasis in the body [25]. Conventional antibodies are characterized by their distinctive Y-shaped structure, composed of two identical heavy chains and two identical light chains, which have been meticulously engineered to ensure exceptional efficiency in antigen binding and neutralization. pAbs represent one of the earliest forms of antibodies to be investigated. They are typically acquired by administering an antigen to an animal, such as rabbits or sheep, which elicits an immune response; subsequently, the antibodies are isolated and purified from the animal’s serum. Due to the presence of multiple B-cell clones in the animal, each producing antibodies against different epitopes of the antigen, the antibody mixtures obtained through this method are referred to as pAbs. While pAbs play a significant role in early biomedical research, their complex composition, low specificity, and large batch-to-batch variability limit their use in high-precision experiments and clinical applications [26].

A major breakthrough in the development of antibody technology occurred in the 20th century with the introduction of mAb technology by Köhler and Milstein, who pioneered the “continuous culture of fusion cells secreting antibodies of predetermined specificity” [27]. This technology was developed using the hybridoma technique. The successful immortalization of mouse cell lines that secrete a single type of antibody with unique antigenic specificity achieved using hybridoma technology has significantly propelled the field of antibody research and application. This technology facilitates virtually unlimited production of pure, highly specific mAbs in vitro. However, mAbs encounter several challenges in large-scale applications, including high production costs associated with mammalian cell expression systems, difficulties in optimizing antibody performance through genetic engineering, and immunogenicity concerns that may arise from using mouse antibodies in human therapeutic applications [28]. These technical bottlenecks have prompted scientists to explore new antibody forms and sources.

The foundational discovery that established the basis for camel antibody technology dates back to the early 1990s, when Hamers-Casterman identified a novel light-chain-deficient antibody in camels [19]. These heavy-chain-only antibodies consist of two constant structural domains, a hinge region, and a variable domain of the heavy chain of the heavy-chain antibody, also known as an Nb. The variable heavy-chain structural domain of these antibodies is capable of binding antigens and represents the smallest functional fragment derived from naturally occurring immunoglobulins. Due to the absence of the first constant structural domain (CH1), which has a lower molecular weight than its conventional counterpart, the molecular weight of the Nb is approximately 15 kDa [29]. In contrast to conventional mAbs and pAbs, the antigen-binding sites of HcAbs found in camelids are exclusively formed by VHHs. This distinctive structure endows VHHs with exceptional stability and antigen-binding capabilities, facilitating genetic engineering for effective recombinant production [21]. Furthermore, other animals exhibit strategies analogous to those of antibody diversification. For instance, sharks produce an antibody known as IgNAR (the variable structural domain of the immunoglobulin neoantigen receptor), which, like Nbs, is both small and stable [20]. In 2018, the world’s first Nb therapeutic, Cablivi^®^ (caplacizumab), produced by Ablynx (subsequently acquired by Sanofi), was authorized by the European Union for the treatment of acquired thrombotic thrombocytopenic purpura in adults, marking a significant step toward the commercialization of Nbs as a new class of antibodies [30]. The product’s global sales reached EUR 211 million in 2022, underscoring the substantial market potential and clinical significance of Nb technologies.

### 2.2. Unique Properties of Nbs

As the star of antibodies, Nbs have natural advantages over mAbs and pAbs, and have great potential for development in the field of testing.

#### 2.2.1. High Stability and Solubility

Since Nbs consist of only one heavy chain, Nbs exhibit resistance to pH fluctuations and proteolytic resistance [21]. Nanobodies contain cysteine residues in their complementarity-determining regions (CDR1 and CDR3), which facilitate the formation of disulfide bonds, enhancing their structural stability. Disulfide bonds contribute to a more stable conformation of Nbs, rendering them less susceptible to denaturation by external factors such as temperature, organic solvents, and acidic or basic environments. The folding of the CDR3 ring and the hydrophilicity of the framework-2 region give them high solubility and low aggregation in aqueous solutions [31]. Notably, the thermal resilience of Nbs surpasses conventional antibodies, with studies reporting full binding retention after 7 days at 37 °C and 40–80% activity preservation after prolonged 90 °C incubation [32], whereas traditional antibodies under identical conditions retain ~20% activity [33].

#### 2.2.2. Low Immunogenicity

Given their relatively small size and simple structure, Nbs exhibit low immunogenicity, reducing the likelihood of stimulating an immune response [34]. Furthermore, the gene sequences of Nbs share a high degree of homology (67–87%) with the human VH gene family 3, which further enhances their biocompatibility; this similarity reduces the likelihood of triggering an immune response in humans [35]. In addition, conventional antibodies contain a fragment crystallizable (Fc) portion that can trigger complementary effects and cell-mediated immune responses. However, Nbs consist solely of VHH and do not contain Fc fragments. As a result, Nbs do not trigger immune responses elicited by Fc fragments, thereby reducing their immunogenicity [36].

#### 2.2.3. High Specificity and Antigenic Affinity

The heightened affinity of Nbs compared to conventional antibodies (e.g., human or murine-derived antibodies) is primarily attributed to their precise antigen-specific recognition. Nbs consist of four conserved sequences and contain three CDRs. Notably, the CDR3 of Nbs comprises 16–18 amino acid residues, significantly exceeding the counts found in human and murine VH (12 and 9 residues, respectively). This region can adopt a convex ring structure that facilitates antigen binding. The compact size of Nbs enables them to engage with elusive epitopes, which are typically challenging for conventional antibodies to recognize. This characteristic not only enhances the Nb’s ability to bind antigens but also allows for interactions with some difficult epitopes that are generally less recognizable by traditional antibodies [37].

#### 2.2.4. Ease of Expression and Production

MAbs are large multimeric proteins that commonly undergo post-translational modifications. Consequently, their production is restricted to eukaryotic systems, necessitating extensive use of large-scale mammalian cell cultures along with prolonged screening and purification processes. These factors lead to significantly higher production costs [38]. In contrast, Nbs present a favorable alternative for reducing mAb production costs. Nbs lack glycosylation modifications, which enable recombinant production within prokaryotic expression systems. Expression levels in *E. coli* can vary from 1 to 100 mg/L, and can be substantially increased by optimizing expression conditions, such as temperature, pH, and the composition of the culture medium [39].

#### 2.2.5. Ease of Manual Modification and Optimization

Another advantage of Nbs is the single-domain structure, which simplifies molecular design. Nbs are composed of only heavy-chain variable regions (~15 kDa), lacking the light chains and complex hinge regions of traditional IgGs, making them compact and free of redundant regions. This single-domain property allows them to be used as a “molecular building block” that can be genetically engineered to flexibly link multiple VHH domains to generate various forms of Nbs. For instance, Nbs can be engineered as bivalent (e.g., through dimerization), bicomplementary (targeting two domains of the same antigen), or bispecific designs (targeting two distinct antigens) to enhance their affinity or redirect their specificity [40,41]. This characteristic simplifies the process of manual manipulation and optimization, making it more efficient than previously possible, and is ideally suited for diverse applications. Moreover, Nbs can be fused with tags, including His tags and fluorescent tags such as green fluorescent protein (GFP), enabling customized optimization strategies for various research domains [42].

## 3. Immunoassay and Immunosensor

To date, tens of thousands of articles have been written about immunoassays and immunosensors, and even the concepts of immunoassays and immunosensors are often used together in numerous studies [43], but the links and differences between them are not systematically elucidated, which may mislead readers to some extent [44].

In this section, we wish to revisit the concepts and definitions of immunoassays and immunosensors. They both rely on the specific binding of antigens and antibodies (immune response) to recognize target molecules (e.g., proteins, pathogens, drugs, etc.). But when talking about immunosensors, one should first address their larger scope as biosensors. A biosensor is an analytical instrument that consists of a specific bioreceptor and a transducer [45]. Such bioreceptors as biorecognition components include entities such as enzymes, antibodies, DNA, and aptamers designed to interact with the target analyte in a given sample.

Immunosensors are an attractive branch of biosensors with excellent sensitivity, rapid analytical capabilities, easy pre-treatment procedures, minimal sample volume, simple instrumentation, and versatile applications. When an antigen or its specific antibody is immobilized on the surface of a transducer, the device, called an immunosensor, is an affinity biosensor. Due to the specific binding between the antibody and the corresponding antigen, immunosensors are highly selective and sensitive, making them ideal platforms for several applications, especially in the medical and bioanalytical fields [46].

In the field of immunosensors, antibodies act as recognition components, and subsequent converters convert the recognition process into measurable signals for analysis. They are categorized according to their conversion method and include various types, such as electrochemical, thermal, fluorescent, electrochemiluminescent, optical, and piezoelectric biosensors [47]. The sensitivity of immunosensors, on the other hand, is highly dependent on the selectivity and affinity of antibodies to form stable immune complexes. Factors such as antibody preparation procedures, advances in immobilization technology, and the integration of labeling methods and high-performance conversion methods also significantly affect the bioanalytical performance of immunosensors [48], which are more oriented towards devices or appliances, and are more fully integrated systems [49].

In contrast, immunoassays are highly selective bioanalytical methods that use antibodies or antigens as biorecognition agents to measure the presence or concentration of analytes in solution [50]. Immunoassays come in a variety of formats, including LFIA, ELISA, surface-enhanced Raman scattering (SERS) immunoassay, surface plasmon resonance (SPR) immunoassay, magnetic immunoassay, fluorescence immunoassay, electrochemical immunoassay, and electrochemiluminescence immunoassay. Despite the different formats, immunoassays have similar structures and procedures. First, a substrate is required to build an assay platform capable of executing an immune response. Immunorecognition elements (including antibodies and antigens) interact specifically on the substrate platform [51]. After target recognition, a signal transducer converts the antibody–antigen interaction into an output signal by reacting with other substances. Alternatively, the signaling probe outputs the detection signal directly through its intrinsic properties without any conversion.

## 4. Nb-Based Immunoassay Applications in Detecting Food Hazards

Nbs represent a new generation of biorecognition tools, characterized by their nanoscale size and exceptional specificity, enabling accurate recognition of substances such as bacteria, viruses, and toxins in food. These recombinant Nbs are engineered for use as primary recognition elements in food safety detection systems. Their development involves the immunization of camelids (e.g., llamas or alpacas) or sharks to generate antigen-specific VHH libraries, followed by phage display screening to isolate high-affinity clones. Selected Nbs are typically expressed in microbial systems (e.g., *E. coli*) and may undergo sequence optimization (e.g., codon usage and stability-enhancing mutations) or functionalization (e.g., biotinylation and fluorescent labeling) to enhance compatibility with detection platforms. Upon binding to the target substance, they are converted into a quantifiable signal output by various mechanisms (including optical, electrochemical, and fluorescent methods), enabling rapid and sensitive detection of food hazards. In recent years, Nb-based immunoassay technologies have been rapidly developed, offering a fast and efficient approach for analyzing biotoxins, bacteria, pesticide residues, and allergens. The widespread adoption of Nb-based immunoassays has shown promise in advancing highly sensitive and stable signal amplification techniques for detecting food hazards

### 4.1. Detection of Biotoxins

Biotoxins are naturally occurring compounds produced by microorganisms, plants, and animals that pose a potential threat to human health, causing a wide range of negative effects upon exposure. Common categories of biotoxins include bacterial, marine, and mycotoxins, each distinguished by distinct properties and associated health risks [52]. Biotoxins can enter the human body through ingestion, inhalation, or contact with contaminated substrates, leading to a range of clinical manifestations depending on the type of toxin and the level of exposure. Mycotoxin contamination commonly occurs during the harvest, processing, storage, and transportation of agricultural commodities. For instance, aflatoxins, which are readily produced during the growth of cereals such as maize, wheat, and rice, are particularly toxic, mutagenic, and carcinogenic [53]. Ochratoxin A (OTA) and deoxynivalenol (DON) are found in a wide range of cereal grains, including wheat, maize, barley, rice, oats, sorghum, and rye, and have been shown to be carcinogenic, nephrotoxic, and hepatotoxic [54]. Marine toxins can be further classified based on their carriers, including shellfish toxins, ciguatoxins, tetrodotoxins, mussel toxins, and diarrheal shellfish toxins, which are common in Chinese coastal waters [55]. These marine toxins can enter the food chain and cause poisoning in humans, which may be fatal in extreme cases. For instance, microcystins can damage the liver, kidneys, adrenal glands, and stomach, interfere with the nervous system, and induce cancer [56]. Furthermore, bacterial toxins, another major class of biotoxins, can cause food poisoning by inhibiting protein synthesis, leading to neurotoxicity. Specifically, staphylococcal enterotoxins can cause sudden symptoms, including vomiting, abdominal pain, and stomach cramps [57]. Although biotoxins are typically present at low levels in food, their risk is difficult to control effectively and poses a multifaceted threat to food safety. Therefore, developing rapid and sensitive methods to detect biotoxins is essential. Currently, high-affinity Nbs targeting a wide range of substances have been developed for use in highly sensitive immunoassays for biotoxin detection, resulting in significant breakthroughs in this field.

ELISA is widely employed in biotoxin detection due to its advantages of low cost, high sensitivity, and ease of operation [58]. For instance, Wang et al. [59] selected specific Nbs against iso-tenuazonic acid (ITeA) using phage display technology, elucidated the recognition mechanism of Nb-ITeA through molecular simulations, and developed an indirect competitive ELISA (ic-ELISA), achieving a limit of detection (LOD) of 0.09 ng/mL. In another work, an ic-ELISA based on biotinylated nanobodies (bi-Nbs) was used for the detection of ustilaginidin A [60] and found that the key amino acid sites of Nb-B15 and Nb-C21 binding to ustilaginidin A were mainly located in the FR1 and CDR1 regions. However, the sensitivity of ELISA is relatively low, and the multiple incubation steps involved are time-consuming as well as tedious. To address these challenges, researchers have proposed several innovative strategies. In a recent study, Yan et al. [61] developed a biotin–streptavidin (SA)-amplified ELISA for the determination of aflatoxin B1 (AFB1) by combining the biotin–SA system with SA-labeled polymeric horseradish peroxidase. This system achieved a 3.6-fold increase in sensitivity and a 0.21 ng/mL increase in the IC_50_ value, demonstrating excellent detection performance. Furthermore, Zuo et al. [62] introduced a biotin–SA system and a novel magnetic bead-based ic-ELISA for screening OTA in cereals. This system enables one-step detection of trace amounts of OTA within an assay time of no more than 20 min, achieving a LOD of 0.07 ng/mL and a detection range of 248.8 pg/mL to 5.28 ng/mL. In addition, Zhang et al. [63] synthesized a RANbody with both recognition and catalytic abilities in one step using molecular recombination technology. Based on this, they developed a sandwich ELISA for detecting α-hemolysin, which eliminated the need for secondary and animal-derived antibodies, reduced assay time and cost, and achieved a low LOD of 10 ng/mL under optimal conditions.

**Figure 2:**
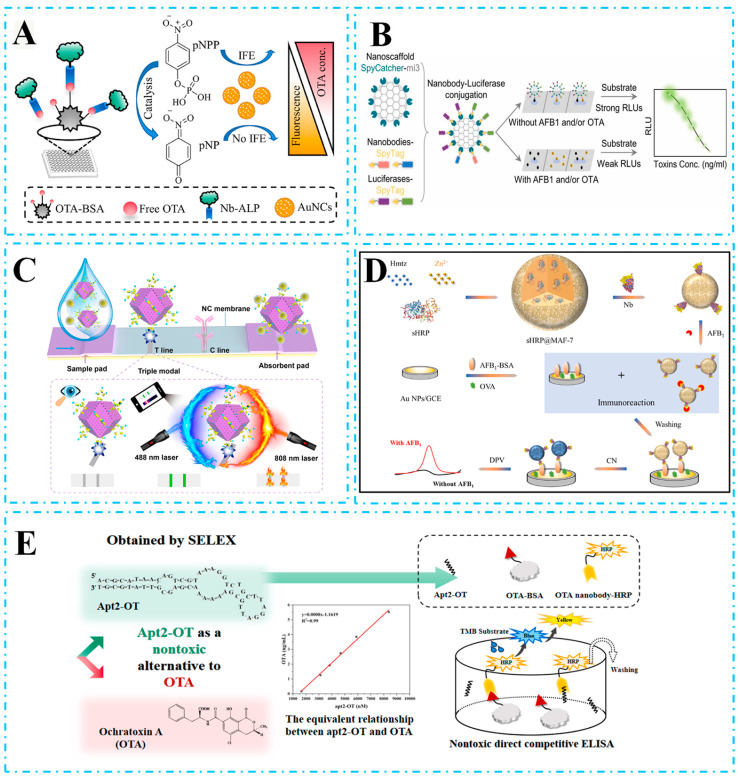
(**A**) Schematic illustration of the IFE-based method using Nb–ALP and AuNCs for OTA detection. (**B**) SpyTag/SpyCatcher enables programmable Nb-luciferase immunofluorescence, and dual-Nb conjugates allow simultaneous AFB1 and OTA detection in one assay. (**C**) Construction of a tLFIA nanoplatform for AFB1 competitive mechanisms for multimodal analysis of AFB1. (**D**) Principle of employing an immunoprobe for the competitive immunosensing of AFB1. (**E**) SELEX for aptamers to bind to the OTA Nb and the principle of nontoxic ELISA.

Traditional ELISA techniques are susceptible to background signal interference, which limits their detection sensitivity. To address this limitation, fluorescence immunoassays (FIAs) are increasingly favored due to their superior sensitivity compared to conventional ELISA methods. Researchers have investigated and introduced a range of fluorescent signals to improve the sensitivity of biotoxin detection assays. Unlike conventional antibodies, the use of genetic engineering to label enzymes onto antibodies offers substantial advantages, with Nbs retaining both antigen-binding capacity and enzyme activity. Alkaline phosphatase (ALP) is a commonly employed enzyme for labeling antibodies in immunoassays. FIA can be established by coupling a phosphate-triggered fluorescence system to an alp-labeled antibody. Inspired by this, Wang et al. [64] developed an Nb28-ALP phosphate fluorescence sensing system. By integrating Nb28-ALP with a phosphate-triggered fluorescence system, they established a highly sensitive and reliable FIA for OTA. Under optimal working conditions, the IC_50_ of the method was 0.46 ng/mL, with a LOD of 0.12 ng/mL. In another work, He et al. [65] utilized an Nb-ALP fusion and AuNC to establish an inner filter effect-based fluorescence immunoassay (IFE-FLIA) for detecting OTA in pepper. As shown in Figure 2A, pNPP significantly inhibits AuNC fluorescence, while ALP catalyzes the hydrolysis of pNPP, thereby enabling the construction of a fluorescence sensing platform for Nb-ALP “on” detection. The inner filter effect between pNPP and AuNCs can quench the fluorescence of AuNCs, and the bifunctional Nb-ALP not only specifically recognizes OTA but also catalyzes pNPP hydrolysis to restore AuNC fluorescence. Under optimal experimental conditions, the IFE-FLIA could be completed in 85 min, with an IC_50_ of 0.22 ng/mL and a LOD of 0.018 μg/kg.

Compared to fluorescence techniques, bioluminescence does not require exogenous light excitation, a characteristic that minimizes interference from background fluorescence and scattered light in complex sample matrices, thereby offering new opportunities for the sensitive and accurate analysis of food and environmental samples [66]. Notably, nanoluciferase (NLuc) is an engineered luciferase derived from Oplophorus, with advantages including enhanced stability, small size (19 kDa), brightness, and continuous luminescence [67]. Inspired by this, Bao et al. [68] successfully developed a one-step bioluminescent enzyme-linked immunosorbent assay (BLEIA) for the determination of OTA in coffee samples by preparing an Nb-Nluc fusion protein. This method allows for one-step incubation and detection by replacing the substrate 3,3′,5,5′-tetramethylbenzidine (TMB) with the luciferase substrate furimazine, achieving a limit of detection (LOD) of 3.7 ng/mL under optimal conditions. In another study, Wang et al. [69] fused isolated Nb-3f9 with Nluc for the detection of streptococcal toxin and TeA and developed two luminescence strategies, CLEIA and BLEIA, achieving detection limits of 0.3 ng/mL and 1.1 ng/mL. However, the inherent 1:1 Nb:Nluc stoichiometry in Nb-Nluc-based BLEIA further limits the maximum achievable signal amplification and sensitivity and lacks multiplexed detection capabilities. In a recent work, as shown in Figure 2B, the proposed nanoscaffold-based BLEIA enables the tunable coupling of nanobodies to luciferase by using SpyTag/SpyCatcher junctions to programmatically attach nanobodies and luciferase to 60-Meric protein nanoscaffolds, enabling the simultaneous detection of AFB1 and OTA [70]. This method significantly improved sensitivity compared to conventional methods, achieving multiplexed detection for AFB1 with a LOD of 0.251 ng/mL and OTA with a LOD of 0.113 ng/mL.

LFIA has been developed as a complementary tool to instrumental analysis due to its ease of operation, low cost, simple production, and reduced time requirements. For example, Pang et al. [71] developed Nb-based LFIA using gold nanoflowers (AuNFs) for the rapid detection of AFB1. By electrostatic adsorption, AuNFs-anti-G8-DIG Nbs were used as probes, achieving a LOD of 0.1 ng/mL under optimal conditions. Conventional LFIAs typically rely on a nondirectional coupling strategy between antibodies and tracer materials, which can impair the functionality of Nbs with low molecular weights [72]. Notably, Wang et al. [73] created an Nb-avi/SA@QD probe using an Avi-tag/strept affinity element-directed coupling strategy, which greatly preserved the detection sensitivity and stability of the probe. And the LFIA method built in this way achieved a detection limit of 0.095 ng/mL and a visual cutoff level of 1.25 ng/mL for AFB1 under optimal conditions. Furthermore, multi-modal detection strategies not only reduce analytical data fluctuation in complex sample matrices but also promote mutual validation between different detection modes, significantly enhancing the accuracy, reliability, and diversity of detection [74]. Wu et al. [75] synthesized photothermal material gold core–petal nanoparticles by a polydopamine-assisted two-step method and prepared a photothermal LFIA using Nbs, which was a strategy to improve the stability of the LFIA by using the Nbs as a “tolerant umbrella”, and achieved a LOD of 1.68 ng/mL in colorimetric mode and a LOD of 0.58 ng/mL in photothermal mode. In addition, Li et al. [76] proposed a colorimetric, fluorescent, and photothermal lateral flow immunoassay based on self-assembled multivalent Nbs (Figure 2C). The multivalent nanoantibodies improved their performance, and the metal–organic carbon nanomaterial Zn-CN was chosen to synthesize the Zn-CN@Nb26-EGFP-H6 multifunctional probe by electrostatic adsorption coupling due to its advantages of large specific surface area and porous structure. The triple signals generated had detection limits of 0.0012, 0.0094, and 0.252 ng/mL, with sensitivities 628-fold and 42-fold higher than those of the original nb26-based ELISA and single-mode LFIA, respectively.

Immunosensors combine the advantages of immunoassays and biosensors, such as rapidity, sensitivity, and precision, making them promising tools for food and environmental analyses. A variety of immunosensors have been developed for biotoxin detection, often in combination with Nbs [77,78]. Due to the small size and single structural domain of Nbs, it is challenging to label signal reporter genes onto Nbs, which results in low signal densities in immunoassays. Liao et al. [79] proposed integrating nanobodies with biomimetic mineralized metal–organic frameworks (MOFs) to overcome the drawback of Nbs’ difficulty in carrying signal reporter molecules. As shown in Figure 2D, a large amount of succinylated horseradish peroxidase (sHRP) was encapsulated in a single MOF, and the mineralized MOF protected the sHRP from denaturation, and the prepared immunoprobe catalyzed the production of precipitation from 4-chloro-1-naphthol, which led to the amplification of the detection signal, and achieved a limit of detection of 20.0 fg/mL for AFB1 and a response in the range of 50.0 fg/mL~20.0 ng/mL. Furthermore, Tang et al. [80] proposed a quantum dot-based Nb-mediated Fourier resonance energy transfer (FRET) immunosensor for OTA detection. Due to the small particle size of Nbs, which shortens the effective FRET distance and improves efficiency, the sensor can complete detection within 5 min and achieves a lowest LOD of 5 pg/mL. This homogeneous approach not only shortens the test time but also improves detection sensitivity.

In the field of immunological analysis of biotoxins, nontoxic immunoassay techniques are increasingly becoming a focus of research due to the operational risks and environmental contamination associated with the use of highly toxic antigens as standards. Anti-unique antibodies (AIds), a class of potential alternatives, have attracted attention because of their ability to specifically bind to the region where the antibody interacts with the antigen, mimicking the three-dimensional structural function of the antigen [81]. Wang et al. [82] successfully obtained three specific β-type AId-Nbs targeting the internal structure of TeA mycotoxin through immunophage display library screening, and selected the optimal performing AId-Nb2D to construct the Nluc-functionalized fusion monomers. In another study, Cai et al. [83] developed a nontoxic enzyme immunoassay for AFM1 in milk and dairy products using the anti-unique VHH C4 as a surrogate standard, and the proposed enzyme immunoassay had a lower LOD of 0.035 ng/mL. However, it is important to note that screening for AIds is costly and time-consuming, which limits its broader application. Therefore, researchers have begun to explore alternatives to chemically synthesized artificial antigens [84]. Mimetic peptides, which bind to the antigen-binding site of a specific target antibody screened using phage display technology, are favored for their ability to mimic antigenic epitopes. In immunoassays, peptidomimetic peptides have been used as substitutes for competing antigens or reporter ligands, significantly simplifying the detection process [85]. Yang et al. [86] screened peptidomimetic peptides against OTA from phage-displayed peptide libraries using Nb beads, and constructed two peptidomimetic peptides, namely PN-ELISA and APN-ELISA, which are specific to the target antigen-binding site of the peptide. The strategy achieved LOD of 0.014 ng/mL and 0.027 ng/mL, providing a powerful tool for sensitive detection of OTA. Nevertheless, phage display peptides are often challenging to screen due to the presence of host–cell toxicity forces, which increases the difficulty of screening. Thus, there is a need for new alternatives that offer short preparation times and minimal batch variation. Hou et al. [87] used aptamers as a competitive alternative toxin standard and screened OTA-specific apt2-OT from a DNA library containing a 36-nucleotide random region. As shown in Figure 2E, the unmodified aptamer competed with the OTA-BSA conjugate for the binding site of the OTA-Nb–biotin conjugate. HRP-SA was then added, and the final signal was monitored via the TMB color rendering reaction, with higher-affinity aptamers producing a lower color signal. As an OTA standard, this aptamer has a LOD of 0.23 ng/mL in nontoxic direct competitive ELISA, providing a new strategy and direction for nontoxic immunoassay of biotoxins.

Table 1 summarizes recently reported Nb-based immunoassay strategies for the detection of biotoxins. Numerous studies have demonstrated the development of high-affinity Nbs for biotoxin detection, which are combined with various output formats (e.g., fluorescence, bioluminescence, etc.) to enable highly sensitive immunoassays.

### 4.2. Detection of Foodborne Pathogens

Foodborne illnesses resulting from food contamination represent a significant global public health challenge, with their associated high morbidity, mortality, and substantial economic burden impacting various aspects of society [95]. According to 2011 data from the Centers for Disease Control and Prevention (CDC), approximately 48 million individuals in the United States contract foodborne illnesses annually, with 320,000 requiring hospitalization and up to 3000 dying as a result [1,96]. Notably, 29% of these cases are directly linked to contaminated food [3,97]. The causes of foodborne illnesses are generally understood to stem primarily from the consumption of food or water contaminated with pathogens or their toxins. The microorganisms responsible for foodborne illnesses are termed foodborne pathogens, encompassing a broad range of organisms, including bacteria, viruses, fungi, and parasites [98]. Further analysis reveals that certain foodborne pathogens are commonly linked to large-scale outbreaks of foodborne illness, including *Salmonella*, *Listeria*, *E. coli*, *Vibrio parahaemolyticus*, and so on [99]. These pathogens present a significant threat to public health through various pathways of contamination within the food chain. For instance, *Staphylococcus aureus* can cause skin infections, pneumonia, and even sepsis [100]. *Salmonella* causes both sporadic and widespread gastrointestinal diseases [101]. *Vibrio parahaemolyticus* can cause wound infections, ear infections, or sepsis [102]. This shows the urgency of rapid detection of foodborne pathogens [103]. As immunoassay techniques continue to advance, their application in detecting foodborne pathogenic bacteria has become increasingly widespread and intensive. These techniques enable the efficient and accurate detection of pathogenic microorganisms in food samples, such as meat, seafood, and fresh vegetables, providing critical support for the timely identification and response to potential foodborne disease threats. However, traditional polyclonal and monoclonal antibody-based immunodetection methods, despite their advantages, face several challenges. One such challenge is the difficulty in detecting *Staphylococcus aureus* due to unwanted interactions between immunoglobulin (Ig)-binding proteins on *S. aureus* and the crystallizable fragment (Fc) region of the antibody, which can lead to false-positive signals [104]. Additionally, the reproducibility crisis concerning antibodies has emerged as a widespread challenge in both academia and industry, and was extensively discussed in a series of articles in *Nature* in 2015 [105]. The absence of Fc-terminated Nbs and their comprehensive characterization could pave the way for the development of novel immunoassays, thereby expanding the scope of detection for various foodborne pathogens [106].

**Figure 3:**
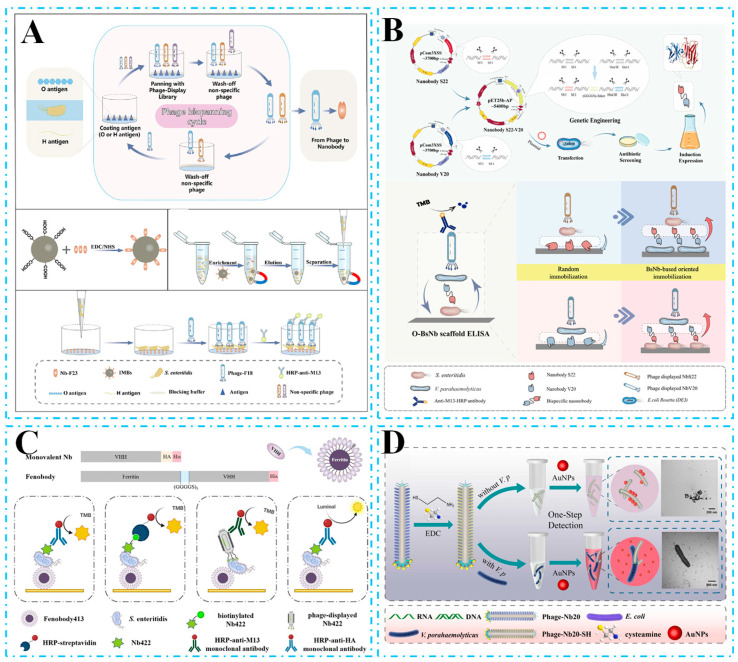
(**A**) Sandwich ELISA for the detection of *S. enteritidis* after enrichment. Target bacteria from a food sample were isolated by the specific nanobody-based immuno-magnetic beads. Then, the enriched S. enteritidis was eluted and detected by double-nanobody sandwich ELISA. (**B**) Oriented immobilization sandwich ELISA for *V. parahaemolyticus* and *S. enteritidis* detection by BsNb. (**C**) Brief schematic diagram for construction of the fenobody and the schematic comparison of four sandwich ELISA modes for *S. enteritidis* detection. (**D**) Construction of a one-step colorimetric immunosensor based on a thiolated phage-displaying Nb for *V. parahaemolyticus* detection. Thiolation of phage–Nbs by EDC chemistry and the proposed colorimetric method for V. parahaemolyticus detection.

In ELISA, He et al. [107] isolated the first Nb against *Salmonella enteritidis* and developed a sandwich ELISA method using Nb13 as the detection antibody, achieving a LOD of 1.4 × 10^5^ CFU/mL. In another work, Hu et al. [108] developed a *Staphylococcus aureus* sandwich ELISA using Nbs that eliminated unnecessary interactions between the Fc region and igg-binding proteins. The sandwich ELISA for Nb147 and biotinylated Nb147 was used for the capture and detection of *S. aureus*, achieving a LOD of 1.4 × 10^5^ CFU/mL. To minimize interference from food substrates, shorten the enrichment period, and enhance detection sensitivity, Bai et al. [109] developed a dual Nb-based sandwich ELISA coupled with immunomagnetic separation for the rapid enrichment and detection of *Salmonella enteritidis*. As shown in Figure 3A, they used an epitope-based bioscreening approach targeting O and H antigens to isolate Nbs specific for *Salmonella enteritidis*, thus avoiding the trial-and-error approach of pairwise Nb selection. The strategy was able to detect 1 CFU of *Salmonella enterica* within 4 h.

However, when Nbs are used for the detection of biological targets such as bacteria or macromolecules (e.g., proteins), their unique structural properties result in binding interfaces occupying a large proportion of the total surface area, which often leads to the masking of the binding sites and at the same time raises the issue of a reduced signal-to-noise ratio and random passive adsorption on physical surfaces. This severely limits the functional integrity of Nbs as a detection element, especially in the construction of a highly sensitive bi-Nb sandwich ELISA [110]. To addressed this issue, Ren et al. [111] utilized soluble Nbs as capture elements in combination with phage-displayed Nbs as detection probes, developing an innovative self-paired sandwich ELISA strategy capable of simultaneously detecting five *Salmonella serotypes*. In this strategy, SA serves as a scaffold to immobilize biotinylated Nbs in a targeted manner, effectively circumventing the negative impacts of passive adsorption. The SAB-ELISA system reduced the detection time to 180 min and achieved a LOD as low as 4.23 to 9.15 × 10^3^ CFU/mL, demonstrating both its excellent analytical performance and broad applicability for detecting various analytes. In another work, Wang et al. [112] introduced a novel concept for an organism immobilization platform, leveraging the unique properties of bispecific Nbs to design bacterial organism–Nb complexes as multifunctional “bioscaffolds”. This design facilitates the targeted immobilization of BsNb while also utilizing its function as a capture antibody, significantly enhancing the flexibility of detection strategies. As shown in Figure 3B, inactivated *Vibrio parahaemolyticus* and *Salmonella enterica* were employed as “bioscaffold” components to construct a double-sandwich immobilization ELISA platform. Inactivated BsNb was detected against *Salmonella enterica* at 3.33 × 10^3^ CFU/mL and *Vibrio parahaemolyticus* at 6.35 × 10^3^ CFU/mL. Additionally, phage display technology is an effective approach to compensating for the affinity defects of Nbs [113]. Zhang et al. [114] developed a phage-mediated sandwich ELISA for *Cronobacter sakazakii*. In this strategy, luminol is used to replace TMB, and the signal generation is optimized to reach 1.04 × 10^4^ CFU/mL.

Polymerization of Nbs can enhance the efficiency of immobilization by increasing the volume of Nbs, thereby generating additional antigen-binding sites [115]. Meanwhile, the advantages of excellent thermal stability and high specificity of monovalent Nbs can be maintained through genetic modification or chemical coupling. Inspired by this, Liao et al. [116] designed homodimeric and heterodimeric Nbs based on Nb413 and Nb422, and established a sandwich ELISA demonstrating the use of bivalent Nbs and phages, with a LOD of 2.364 × 10^3^ CFU/mL. This strategy confirmed that dimeric Nbs exhibit high binding affinity and effectively reduce the occupancy of immobilized antibody-binding sites. In another study, ferritin display technology offers an efficient Nb polymerization strategy, enabling self-assembly into 24-valent Nb polymers through genetic or chemical coupling of ferritin and Nb [117]. Liao et al. [118] constructed a ferritin-Nb fusion and designing three sandwich ELISA modes for detecting *Salmonella*. This method is shown in Figure 3C. The constructed Fb formed a self-assembled 24-valent nanocage structure, enhancing the affinity 35-fold while maintaining superior thermal stability and specificity compared to conventional Nbs. By replacing the TMB chromogenic substrate with luminol, the developed FbNb-CLISA achieved a LOD of 2.94 × 10^3^ CFU/mL, confirming that ferritin display technology is a promising approach to expand the applicability of Nbs in food testing and other areas requiring multivalent modifications.

The research community continues to seek the development of simpler, faster, more powerful, and sensitive bacterial detection strategies. Colorimetric biosensors have garnered significant attention in the field of food contaminant analysis due to their intuitive visual readout, portability, and cost-effectiveness. Wang et al. [119] proposed a one-step, label-free colorimetric strategy for detecting *Vibrio parahaemolyticus* using M13 phage-displaying Nbs. As shown in Figure 3D, sulfation of phage–Nbs on pVIII chitin induced the aggregation of AuNPs, which was inhibited by specific interactions between the Nbs and bacteria. This interaction caused a change in the plasmon resonance properties of the surface, leading to a visible color change. Detection using this colorimetric immunosensor was completed within 100 min, with a visual LOD of 10^4^ CFU/mL and a quantitative LOD of 10^3^ CFU/mL. In addition, Zhang et al. [120] further expanded the application of colorimetric sensing technology. They modified AuNPs on a 3D KMO surface to improve the photothermal conversion efficiency of KMO@Au photothermal sensing probes, and effectively coupled a flower-like 3D KMO@Au photothermal agent by using small-sized Nbs. By integrating colorimetric and photothermal sensing mechanisms, the constructed Nb-DITS achieves a dual-mode quantitative detection of *Salmonella typhimurium*, with a LOD of 10^4^ CFU/mL in colorimetric mode and 10^3^ CFU/mL in photothermal mode.

Table 2 summarizes recently reported Nb-based immunoassay strategies for detecting foodborne pathogens. Ranging from traditional ELISA methods to emerging colorimetric biosensors, these innovations not only simplify the detection process and shorten detection time but also significantly enhance sensitivity and accuracy, providing robust technical support for the prevention and control of foodborne pathogens. However, because most tested samples consist of meat, eggs, milk, and other food matrices, Nb-based immunoassays for detecting foodborne pathogens face challenges such as matrix interference and prolonged detection times. Additionally, overcoming hidden binding sites and reducing the signal-to-noise ratio in the detection of large molecules are critical areas for future research and development.

### 4.3. Detection of Pesticide Residues

Pesticides are chemicals used for controlling pests, including rodents, insects, fungi, and weeds. Depending on the target organism, pesticides can be classified as rodenticides, insecticides, fungicides, or herbicides. The use of pesticides in modern agriculture has increased to control pests and ensure food security. Global pesticide consumption in 2019 reached approximately 4.19 million tons, with China being the largest consumer (1.76 million tons), followed by the United States (40.8 million tons), Brazil (377.7 thousand tons), and Argentina (204.4 thousand tons) [123]. However, improper and excessive pesticide use has resulted in significant pesticide residues in both food and the environment [124]. The misuse of pesticides can result in the formation of residues (parent compounds and/or active degradation products) that may appear in processed commodities and ultimately enter the food chain [125]. Pesticide residues and their metabolites can be transferred through the food chain via enrichment and bioaccumulation, affecting the quality and safety of agricultural products, jeopardizing the environment, and endangering human health. For example, the widespread use of dicamba poses a direct risk to human health, with agricultural health studies indicating an association between dicamba use and colon and lung cancers [126]. The extensive and inappropriate use of quinphos poses significant threats to the environment, food safety, and human health [127]. Putrescine (PRM) may cause chronic toxic effects and severe kidney damage [128]. Therefore, there is a need to develop rapid techniques for detecting pesticide residues, among which antibody-based immunoassays have been developed and approved as standard screening methods [129]. Among these, Nbs have been widely used for detecting small-molecule pesticide contaminants due to their superior stability and extended storage capacity.

In ELISA, Xu et al. [130] obtained Nbs against cyanobenzamide and chlorpyrifos through alpaca immunization and phage display library construction, and developed an Nb-based ELISA for detecting these two insecticides. Wang et al. [131] successfully obtained Nbs specifically recognizing dicamba by immunizing camels and constructing a phage display library. They developed an ic-ELISA based on Nb-242, which achieved an IC_50_ of 0.93 μg/mL with a linear range of 0.11–8.01 μg/mL under optimal conditions. Additionally, they identified the key amino acids in Nb-242 that bind to dicamba through homology modeling and molecular docking. In another work, Liu et al. [132] prepared several bispecific Nbs with different lengths of hydrophilic linker groups and attachment sites, and constructed a bispecific brief-joining ELISA by selecting a BsNb with good stability and sensitivity, achieving LOD of 0.8 ng/mL for carbaryl and 0.4 ng/mL for 1-naphthol.

**Figure 4:**
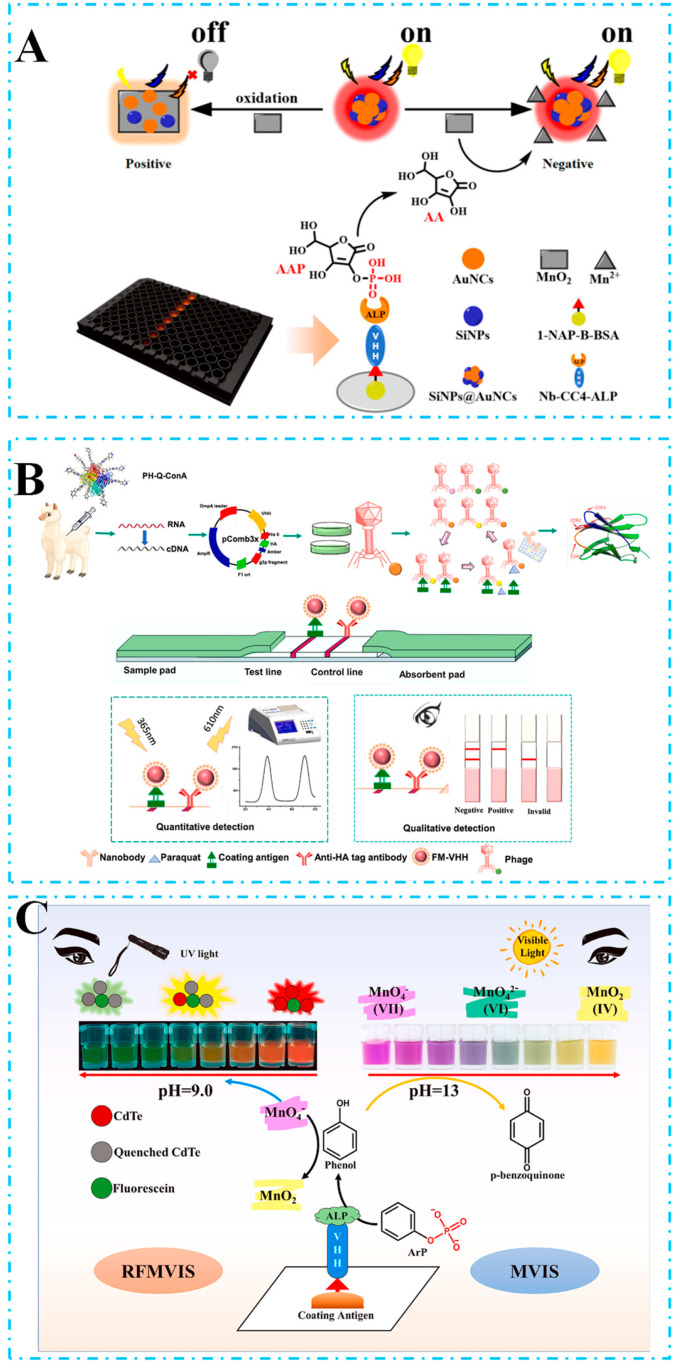
(**A**) A dual-emission system-based ratiometric fluoroimmunoassay (RFIA) for quick and highly sensitive determination of 1-NAP was developed based on a nanobody–alkaline phosphatase (Nb-CC4-ALP) fusion protein. Silicon nanoparticles (SiNPs) were used as an internal reference and for the aggregation-induced emission enhancement (AIEE) of gold nanoclusters (AuNCs), while AuNCs could be quenched by MnO_2_ via oxidation. In the presence of ALP, ascorbic acid phosphate (AAP) can be transformed into ascorbic acid (AA); the latter can etch MnO_2_ to recover the fluorescence of the AuNCs. (**B**) Screening of specific immunogens to immunize alpacas from six designed antigens and screening of highly sensitive nano-antibodies for the construction of TRFICA test strips. TRFICA identifies paraquat, enabling quantitative analysis of test strips and semi-quantitative analysis by the naked eye. (**C**) An anti-fenitrothion nanobody–alkaline phosphatase fusion protein (VHHjd8-ALP) was employed to develop a multicolor visual immunosensor (MVIS) and a ratiometric fluorescence MVIS (RFMVIS). The phenol produced from VHHjd8-ALP can reduce KMnO_4_ directly to achieve MVIS without any extra reaction. Moreover, the KMnO_4_-based regulation chain can be utilized to develop RFMVIS on the basis of rQDs and fluorescein, which further improves the sensitivity.

In FIA, Chen et al. [133] successfully fused VHH with a biotin ligase and an Avi tag, and constructed VHHjd8-BT fusion proteins, which were then coupled with SA-polyHRP for signal amplification. They further synthesized released carbon dots to develop a highly sensitive FIA system, which achieved a LOD as low as 0.03 ng/mL for the target analyte. Furthermore, Lv et al. [134] developed an innovative FIA strategy using gold nanoclusters anchored by Nb-ALP and manganese dioxide (AuNCs-MnO_2_) composites. This strategy modulates the fluorescence reaction through a competitive immunoreaction followed by the alkaline phosphatase-catalyzed generation of ascorbic acid from L-ascorbic acid-2-phosphate, which triggers the decomposition of the AuNCs-MnO_2_ complex and thus achieves a LOD of 5.78 pg/mL for fenitrothion. In another study, Chen et al. [135] prepared well-stabilized Nb-ALP fusion proteins and established a dual-emission system-based fluorescence immunoassay for the ratio 1-NAP analysis method, which showed a high sensitivity of 0.01 ng/mL. As shown in Figure 4A, silicon nanoparticles were used as an internal reference for the aggregation-induced emission enhancement of gold nanoclusters (AuNCs), which can be quenched by MnO_2_ through oxidation. In the presence of ALP, ascorbic acid phosphate is converted to ascorbic acid, which can etch MnO_2_ and restore the fluorescence of the AuNCs. In addition, using fluorescently labeled antibodies as probes not only provides high sensitivity from the enhanced fluorescence signal but also enables non-competitive homogeneous immunoassay to further reduce the LOD.

In LFIA, colloidal gold (CG) has been widely used in ICA owing to its low-cost preparation and easy visual detection. Guo et al. [136] constructed a competitive CG immunochromatographic assay using VHH9 for good detection of parathion in food samples. In addition, Liu et al. [137] biotinylated the obtained NbFM5 and affixed it with SA-labeled AuNPs to preserve the epitope activity and to prevent the decrease in sensitivity due to conventional random electrostatic adsorption. And in this way, a simple and sensitive immunochromatographic assay was developed for the rapid detection of PRM based on biotinylated Nb, achieving a LOD of 0.88 ng/mL. In another work, Zhang et al. [138] developed an ultrasensitive time-resolved fluorescence immunochromatographic assay test strip, which provides a convenient and efficient solution for paraquat detection. As shown in Figure 4B, the test strip utilized polystyrene microspheres encapsulated with time-resolved fluorescent europium(III) [FM] and achieved accurate detection of paraquat by virtue of its unique fluorescence characteristics. The test strip not only supports quantitative analysis using professional readers, but also facilitates semi-quantitative analysis by the naked eye, with a LOD limit as low as 0.0090 ng/mL and an IC_50_ of 0.0588/mL, demonstrating great potential as a low-cost, portable monitoring tool.

In biosensing strategies, Yin et al. [139] developed a sensitive and reliable electrochemical immunosensor based on crosslinked polyvinyl alcohol/citric acid nanofibrous membranes and horseradish peroxidase-labeled antiparathion Nbs for the detection of parathion, with the linear ranges and limits of detection under the optimal conditions of 0.01–100 ng/mL and 2.26 pg/mL, respectively. In addition, multicolor detection is a promising research direction, providing advantages such as a compact design, rapidity, and low cost [140]. Chen et al. [141] developed a multicolor visual immunosensor (MVIS) and a proportional fluorescence MVIS (RFMVIS) using an anti-fenitrothoracin Nb-ALP fusion protein (VHHjd8-ALP) for the detection of fenitrothion. As shown in Figure 4C, after a one-step competitive immunoassay, VHHjd8-ALP bound to the microtiter plate and catalyzed the conversion of the disodium salt of phenyl phosphate to phenol. Under highly alkaline conditions (pH 12), phenol reduces KMnO_4_ to K_2_MnO_4_, which is further reduced to MnO_2_ under alkaline conditions (pH 12), accompanied by a visible violet–green–yellow color shift that can be used for semi-quantitative visual analysis. Compared with the visual detection of ELISA, the multicolor detection of this method is more promising, achieving a LOD of 11.2 ng/mL for MVIS and 7.4 ng/mL for FMVIS.

By combining multiple signal output forms with advanced biosensing strategies, Nbs have emerged as a promising analytical recognition element for agricultural residue detection. Specific innovations are summarized in Table 3.

### 4.4. Detection of Food Allergens

Food allergy is a complex hypersensitivity reaction triggered by specific food proteins and mediated by immunoglobulin E (IgE). It occurs when the body’s immune system abnormally activates in response to exposure to certain foods, leading to clinical symptoms that can range from mild urticaria and gastrointestinal discomfort to severe, life-threatening reactions such as anaphylaxis [143]. Epidemiological data indicate that this condition is widespread, affecting approximately 6–7% of adults and up to 10% of children globally, with a slightly higher prevalence in females [144]. Food allergies not only severely impact the quality of life of affected individuals but also impose a substantial economic burden, with healthcare costs in the United States alone estimated at USD 24.8 billion per year. The prevalence of food allergies continues to rise worldwide [145]. Major food allergens have been identified as proteins, and while allergic reactions have been reported for many foods, more than 90% of food allergies are caused by milk, eggs, fish, crustaceans, peanuts, tree nuts, wheat, and soybeans. Sesame is the ninth most common food allergen, responsible for nearly 90% of all severe food allergy reactions [146]. Given the severity and increasing prevalence of food allergies, the development of efficient diagnostic and management strategies is essential. In this regard, antibody-based immunoassay techniques have become the preferred method for detecting allergenic hazards in complex food matrices due to their ease of use and cost-effectiveness. Conventional immunoassays, which rely on monoclonal or polyclonal antibodies, depend heavily on the quality of purified allergenic proteins and antibodies; the lack of high-quality reagents can lead to inefficient detection. Additionally, identifying suitable antibodies for unidentified allergens can be challenging, highlighting the need for alternative affinity reagents to enhance allergen analysis. By coupling specific antibodies with various substrates (e.g., chromogenic agents, fluorescent probes, and electrochemical sensors), these techniques can generate sensitive and specific detection signals. Nbs are particularly advantageous for food allergen detection due to their unique long CDR3 region, which enables the formation of flexible complementary sites. This allows Nbs to specifically bind to conformational, rather than linear, epitopes of their target antigens, making them a valuable tool for developing efficient detection methods for food allergens [147].

In ELISA, Hu et al. [148] successfully identified β-lactoglobulin (BLG)-specific Nbs and developed a competitive ELISA with a LOD of 4.55 ng/mL. They also investigated the interaction of selected Nbs with BLG-derived peptides through Nb structure modeling and BLG docking, ensuring the detection of intact BLG without interference from hydrolyzed peptides. In another work, Li et al. [149] developed a highly sensitive sandwich ELISA using a specific Nb as the capture antibody, achieving a LOD of 0.24 ng/mL. They explored the mechanism of epitope shielding of the BLG antigen during heat treatment and accurately quantified BLG levels in both pasteurized and UHT milk, successfully distinguishing between the two, thus providing a novel approach for identifying UHT and pasteurized milk. Furthermore, a recent study [150] developed a dual-Nb sandwich ELISA for BLG, with a limit of quantification of 40 pg/mL and an extended linear range of up to 3000 pg/mL, validating its use for the quantitative analysis of BLG in human milk.

**Figure 5:**
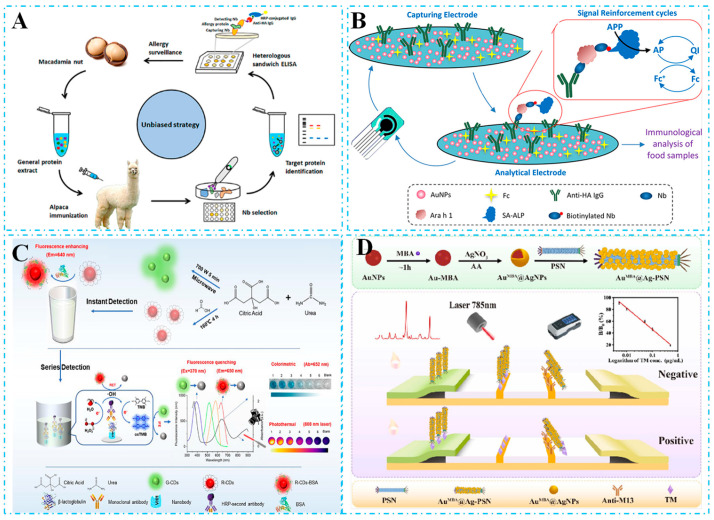
(**A**) Schematic illustration of the selection of Nbs against macadamia allergens based on the unbiased immunization strategy and development of the immunoassay. The work immunized an alpaca with the total protein extracts of macadamia nuts and let the immune response within the animal decide which proteins to be antigenic. (**B**) Schematic illustration of the development of Nb-based electrochemical immunoassay. A carbon paste electrode (CPE) functionalized with Au nanoparticles and anti-HA IgG serves as the capture electrode to immobilize HA-tagged nanobodies; HA-tagged Ara h 1-specific Nbs selectively bind target allergens from complex food matrices; and signal amplification is achieved through sequential binding of biotinylated Nbs, streptavidin–alkaline phosphatase (SA-ALP), and enzymatic redox cycling, enabling ultrasensitive detection via dual catalytic enhancement. (**C**) Schematic of POCT tandem immunosensors. Preparation process of G-CDs, R-CDs, and R-CDs@BSA. A “fluorescence–photothermal” immunosensor based on nanobodies was constructed by introducing the fluorescence signal of R-CDs@BSA and the photothermal signal of oxTMB for the detection of β-lactoglobulin (β-LG). (**D**) Construction of the CM/SERS-LFI using the M13 phage-displayed shark Nb. The SERS tag AuMBA@AgNPs with the Raman signal molecule 4-mercaptobenzoic acid (4-MBA) was prepared and immobilized on the PSN to construct the immunoprobe AuMBA@Ag-PSN. The probe can identify free TM that competes with TM on the T-line, and the optimized CM/SERS-LFI enables quantitative analysis of TM using the probe.

In terms of innovative applications of unbiased immunization strategies, Hu et al. [151] made a significant contribution to the field of food allergen detection by establishing a milestone in the development of these methods. Their study was the first to employ an unbiased immunization strategy for screening Nbs against specific allergens. As shown in Figure 5A, this strategy does not rely on prior knowledge of the allergen, but instead utilizes an extensive immunization and screening process to identify specific antibodies. Using this approach, they successfully screened six Nbs and established a heterologous sandwich ELISA based on the identified Nb pairs. The method demonstrated a good linear response over the range of 0.442–2800 ng/mL, with a low LOD of 27.1 ng/mL. Notably, they extended the application of this unbiased immunization strategy to the detection of peanut allergens. Hu et al. [152] employed peanut universal protein extracts for immunization and used the unbiased selection strategy to identify specific Nbs against peanut allergens, resulting in the establishment of a sandwich ELISA for the detection of Ara h 3, with a LOD of 53.13 ng/mL. This unbiased immunization and selection strategy holds great promise for identifying potential allergens from generic protein extracts and can be applied in analytical assays to screen for allergenic contamination in foods.

In the biosensing strategy, Hu et al. [153] immunized alpacas with Ara h 1 to generate an Nb library targeting Ara h 1. They screened for four specific Nbs and developed an electrochemical immunoassay based on Nbs, constructing capture electrodes integrated with signal-enhancing cycles. As shown in Figure 5B, the electrode surface was coated with AuNPs, and anti-HA IgG was immobilized using an HA-tagged Nb to capture various concentrations of Ara h 1; Ara h 1 was labeled with biotinylated Nb152, which then bound to ALP-coupled SA for signal amplification. This method achieved a linear detection range of 4.5 to 55 ng/mL for Ara h 1, with a LOD of 0.86 ng/mL. In another work, Li et al. [154] developed an Nb-based immunosensor integrating “fluorescence–photothermal” properties for the precise detection of BLG. As shown in Figure 5C, they synthesized water-soluble green and red fluorescent carbon dots from citric acid and urea, respectively. The RCDs exhibited a sensitive fluorescence burst upon reaction with hydroxyl radicals (-OH), achieving a LOD of 0.034 ng/mL in fluorescence mode and 0.075 ng/mL in photothermal mode when used with the Nb platform. Notably, Jiao et al. [155] pioneered the use of phage-displayed shark Nb (PSN) as the foundation for multimodal biomaterials and developed a lateral flow immunosensor that integrates colorimetry and surface-enhanced Raman scattering. This sensor is designed for the competitive detection of crustacean Tropomyosins (TMs), which are assumed to be specific allergens or toxins. As shown in Figure 5D, this design utilizes PSN to anchor AuMBA@AgNP composite nanoparticles, which are modified with the Raman-active molecule 4-mercaptobenzoic acid (4-MBA), forming a Au^MBA^@Ag-PSN probe. This probe specifically recognizes and binds free molecules that compete with TM on the T-line, achieving an ultralow LOD of 0.0026 μg/mL in SERS mode and a sensitivity of 0.0057 μg/mL in colorimetric mode. These advancements significantly enhance the versatility and accuracy of detection methods. This strategy enables both quantitative analysis of TM by measuring the Raman signal intensity of 4-MBA or the color intensity of the T-line and rapid qualitative detection in the field via intuitive color change. These capabilities underscore the great potential of PSN as a multifunctional biomaterial in detection applications.

Despite the growing prevalence of food allergies, effective treatments remain unavailable. Consequently, detection methods must be sensitive, specific, robust, and reproducible. Nbs have gained increasing attention as alternative antibodies for detecting food allergens. Electrochemical, fluorescence, and photothermal immunoassays have been developed as highly sensitive assays, and related innovations are demonstrated in Table 4. In the coming years, the trend will shift towards the development of cost-effective and simplified methods to enable high-throughput screening of foodborne allergens by non-specialists.

## 5. Challenges of Nb-Based Immunoassay from Laboratory to Field

The growing demand for food safety monitoring has positioned immunoassays as prominent analytical tools due to their operational simplicity, rapidity, cost-effectiveness, and adaptability to field deployment. Established methodologies such as ELISA, LFIA, and other immunosensors are widely implemented in food safety analysis. While mAbs and pAbs face limitations in production consistency and ethical concerns regarding animal immunization, Nbs have emerged as promising alternatives to mAbs and pAbs for immunoassay development. While Nb generation initially involves the immunization of camelids (e.g., alpacas), this process aligns with refined ethical practices: camelids experience minimal stress during immunization and blood collection, and a single immunization yields a diverse antibody repertoire without requiring animal euthanasia (unlike hybridoma-based mAb production). Moreover, once the Nb gene is obtained, subsequent production relies entirely on in vitro microbial systems (e.g., *E. coli* or yeast), eliminating the need for further animal use.

However, despite numerous innovative applications of nanobody-based immunoassays documented in research settings, their transition from laboratory prototypes to commercial field applications remains limited. Through a systematic review, three key barriers were identified:

(I) Lack of technological maturity: unlike traditional antibodies, nanobodies usually rely on prokaryotic expression systems (e.g., *E. coli*), which are prone to the formation of inclusion bodies, leading to loss of activity, whereas eukaryotic systems (yeast and mammalian cells), although soluble expression is much better, are expensive (the cost of a single expression on a laboratory scale can be up to 3–5 times that of traditional antibodies).
(II) Lack of standardization: nanobody-based testing methods have not yet formed unified standards and specifications, making it difficult to compare and verify results between different laboratories or companies.
(III) Low market acceptance: users of on-site testing lack understanding of nanobody technology, and the commercial market is still dominated by traditional monoclonal/polyclonal antibodies.

Nanobody immunoassay technology, from the laboratory to the field, needs to cross the “performance reproducibility—equipment portability—cost controllability—universal standards” multiple chasms. Current research should focus more on demand-oriented design and accelerate the implementation of the technology through policy guidance.

## 6. Conclusions and Perspectives

This paper reviews various Nb-based immunoassay strategies for food safety analysis, encompassing nearly all innovative approaches developed in the past five years: (1) preparation of high-performance Nbs and multivalent Nbs, especially dimeric Nbs and fenbodies; (2) development of reporter gene engineered fusion Nbs, including Nb-ALP, Nb-Nluc, and Nb-HRP; (3) creation of novel signaling markers, such as AuNCs, QDs, CPNs, and other nanoparticles; (4) advanced support and carriers for Nb labeling, using materials like MOF, Zn-CNs, and other nanomaterials; (5) development of innovative detection platforms combining fluorescence, bioluminescence, electrochemical, and other strategies; and (6) implementation of multimodal detection by integrating photothermal, fluorescence, and colorimetric methods.

Nb-based immunoassay strategies have revolutionized the rapid detection of common food hazards and allergens, including toxins, foodborne pathogens, pesticide residues, and other allergens. Due to their ultrasmall size, high affinity, specificity, and stability, Nbs, in combination with fluorescence, bioluminescence, colorimetry, surface-enhanced Raman scattering (SERS), and electrochemical detection strategies, have facilitated new approaches in food safety detection, significantly improving detection sensitivity. Numerous examples presented in this review demonstrate the potential of Nbs for highly sensitive and specific detection of food hazards. The core of the Nb immunoassay strategy lies in its molecular recognition ability, which is attributed to the efficient and specific binding between the Nb and its target. By optimizing Nb sequence design combined with advanced genetic engineering techniques, the accuracy and stability of molecular recognition can be further enhanced to meet the demands of contaminant detection in complex food matrices. Additionally, Nbs can be easily chemically modified and surface-functionalized, enabling the construction of multifunctional, highly sensitive immunoassay platforms for rapid on-site detection, thus enhancing the efficiency and scope of food safety regulations.

Overall, Nb-based immunoassays demonstrate significant potential, with future developments expected to enable highly sensitive, accurate, and multiplexed detection, making them ideal for ensuring food safety.

## Figures and Tables

**Table 1:** Recent advances in immunoassay methods for various biotoxins based on Nbs.

Principle	Target	Detection Technique	Detection Label	LOD	IC_50_	Linear Range	Sample	Reference
ELISA	Alternaria mycotoxins tenuazonic acid	IC-ELISA	Nb(B3G3)	0.09 ng/mL	1.3 ng/mL	-	Rice, flour, and bread	[59]
	Ustilaginoidins	IC-ELISA	Nb-B15	-	11.86 µg/mL and 11.22 µg/mL	3.41~19.98 µg/mL and 1.17~32.13 µg/mL	Rice	[60]
	Ochratoxin A	Dc-PEIA	Nb 28	Instrumental LOD: 0.275 ng/mL Visual LOD: 1.56 ng/mL	10.84 ng/mL	5.18~29.32 ng/mL	Black pepper and white pepper	[88]
	Aflatoxin B1	BA-ELISA	Nb 26	0.04 ng/mL	0.21 ng/mL	-	Wheat and corn	[61]
	Ochratoxin A	MBS-ELISA	Nb-2G	0.07 ng/mL	1.17 ng/mL	248.8 pg/mL~5.28 ng/mL	Mung bean, buckwheat, and sorghum rice	[62]
	α-hemolysin	Sandwich ELISA	HLA 39 HLA 17	10 ng/mL	-	10~1000 ng/mL	Milk and pork	[63]
Fluorescence immunoassay	Ochratoxin A	Nb-AP-induced PT-FIA	Nb-AP	0.12 ng/mL	0.46 ng/mL	0.2~1.26 ng/mL	Barley	[64]
	Ochratoxin A	IFE-FLIA	Nb-ALP	0.018 μg/kg	0.22 ng/mL	0.11~0.53 ng/mL	Pepper	[65]
	Staphylococcal enterotoxin B	Dual-mode immunoassay	SEB57 SEB27-vHRRP	Colorimetric mode: 0.12 ng/mL Fluorescence mode: 0.24 ng/mL	-	0.31~2500 ng/mL	Milk, pork	[89]
Bioluminescent immunoassay	Ochratoxin A	BLEIA	Nb 28-Nluc	3.7 ng/mL	-	-	Coffee	[68]
	Tenuazonic acid	CLEIA/BLEIA	Nb39−Nluc	0.3 ng/mL 1.1 ng/mL	8.6 ng/mL 9.3 ng/mL	-	Rice, flour, and apple juice	[69]
	Aflatoxin B1 and ochratoxin A	SA-BLEIA	Nb 28 and Nb 26	AFB1: 0.053 ng/mL OTA: 0.051 ng/mL	AFB1: 0.452 ng/mL OTA: 0.147 ng/mL	-	Cereal powders and spiked cereal	[70]
LFIA	Aflatoxin B1	AuNPs-ICTS	G8-DIG	0.1 ng/mL	5.46 ng/mL	1.02~27.86 ng/mL	Corn	[71]
	Aflatoxin B1	Nb-LFIA	Nb@QD	0.095 ng/mL	0.85 ng/mL	-	Oat	[73]
	Staphylococcal enterotoxin B	NLFIA	anti-SEB Nb7	Colorimetric mode: 1.68 ng/mL Photothermal mode: 0.58 ng/mL	-	1~128 ng/mL	Milk, milk powder, and pork	[75]
	Aflatoxin B1	TLFIA	Nb 26-EGFP-H6	Colorimetric signals: 0.0012 ng/mL Fluorescent signals: 0.0094 ng/mL Photothermal signals: 0.252 ng/mL	-	0.05~100 ng/mL, 0.25~60 ng/mL, and 1~500 ng/mL	Maize	[76]
Immunosensor	Microcystin-LR	Multimodal biosensors	A2.3-SBP	0.26 ng/mL	-	1.0~500 ng/mL	Lake water samples	[77]
	Aflatoxin B1	Fluorescent–colorimetric immunosensor	Nb26-EGFP	0.0024 ng/mL	-	-	Corn	[78]
	Aflatoxin B1	Immunoensor	Nb G8	20.0 fg/mL	-	50.0 fg/mL~20.0 ng/mL	Flour and rice	[79]
	Ochratoxin A	Nb-FRET immunosensor	Nb 28	5 pg/mL	-	-	Rice, oats, barley, and wheat	[80]
	Ochratoxin A	Bioluminescence immunosensor	Nb 28	0.01 ng/mL	0.31 ng/mL	0.04~2.23 ng/mL	Barley, oats, and rice	[90]
Nontoxic immunoassay	Aflatoxin M1	C-ELISA	Nb C4	0.05 ng/mL	0.25 ng/mL	0.10 ng/mL~0.60 ng/mL	Milk, yogurt, and milk powder	[81]
	Tenuazonic acid	BLEIA	AId-Nb NLuc	0.7 ng/mL	6.5 ng/mL	-	Rice, flour, and bread	[82]
	Aflatoxin M1	Toxin-free ELISA	Nb C4	0.035 ng/mL	-	0.045~0.329 ng/mL	Milk and yogurt	[83]
	Aflatoxin M1	Electrochemical immunosensor	Nb 4–1-1	0.09 ng/mL	-	0.25~5.0 ng/mL	Milk	[91]
	Ochratoxin A	APN-ELISA	Nb-C4bpα	0.027 ng/mL	0.169 ng/mL	0.058~0.471 ng/mL	Barley, oats, and rice	[86]
	Ochratoxin A	IC-ELISA	apt 2-OT	0.23 ng/mL	-	0.25~10.50 ng/mL	Flour, corn, and meal	[87]
Enhanced colorimetric enzyme immunoassay	Ochratoxin A	Colorimetric enzyme immunoassay	Nb-ALP-C4bpα	0.018 ng/mL	0.081 ng/mL	0.036~0.175 ng/mL	Barley, oats, and rice	[92]
CLEIA	Aflatoxin B1	MB-CLEIA	Nb-ALP	0.743 pg/mL	0.33 ng/mL	7.23 pg/mL~12.38 ng/mL	Oats, corn, and oil sample	[93]
	Staphylococcal enterotoxin B	Sandwich CLIA	Nb37-ALP	1.44 ng/mL	8.59± 0.37 ng/mL	3.12~50 ng/mL	Pure milk, water, and serum	[94]

**Table 2:** Recent advances in immunoassay methods for various foodborne pathogens have been based on the use of Nbs.

Principle	Target	Detection Technique	Detection Label	LOD	Linear Range	Sample	Reference
ELISA	*Salmonella enteritidis*	Sandwich ELISA	Nb13	1.4 × 10^5^ CFU/mL	-	Whole milk, skimmed milk, and walnut milk	[107]
	*Staphylococcus aureus*	Sandwich ELISA	Nb147 and biotinylated Nb147	1.4 × 10^5^ CFU/mL	10^4^~10^10^ CFU/mL	Milk	[108]
	*Salmonella enteritidis*	IMS-ELISA	Nb-F23	3.2 × 10^3^ CFU/mL	1.4 × 10^4^~5.9 × 10^5^ CFU/mL	Chicken meat, cabbage, tomato, and apple juice	[109]
	*E. coli O157:H7*	Sandwich ELISA	VHH	8.7 × 10^3^ CFU/mL	-	Orange juice, milk, and beef	[121]
	*Salmonella Enteritidis, Salmonella Typhimurium, Salmonella London, Salmonella Paratyphi B, and Salmonella Hadar*	SAB-ELISA	bi-Nb01	6.31 × 10^3^ CFU/mL 9.15 × 10^3^ CFU/mL 4.23 × 10^3^ CFU/mL 7.31 × 10^3^ CFU/mL 7.25 × 10^3^ CFU/mL	-	Milk, honey, pork, and lettuce	[111]
	*Salmonella spp. and V. parahaemolyticus.*	O-ELISA	O-BsNb	*Salmonella* spp.: 3.33 × 10^3^ CFU/mL *V. parahaemolyticus*.: 6.35 × 10^4^ CFU/mL	-	Shrimp and chicken	[112]
CLISA	*Salmonella Typhimurium*	P-CLISA	Nb1 and Nb9	3.63 × 10^3^ CFU/mL	5.1 × 10^3^~1.2 × 10^6^ CFU/mL	Juice, honey, milk, and pork samples	[113]
	*Cronobacter sakazakii*	P-CLISA	Cs-Nb 1 and Cs-Nb 2	1.04 × 10^4^ CFU/mL	-	Milk powder and whole milk	[114]
	*Salmonella*	BNb-ELISA	Nb413 and Nb422	2.364 × 10^3^ CFU/mL	-	Ham sausage, beef, and shrimp	[116]
	*S. Enteritidis*	FbNb-ELISA FbBio-ELISA FbP-ELISA FbNb-CLISA	Nb422 and biotinyiated Nb422	3.56 × 10^4^ CFU/mL 5.83 × 10^5^ CFU/mL 4.42 × 10^5^ CFU/mL 2.94 × 10^3^ CFU/mL	-	Juice, ham sausage, and honey	[118]
Immunosensor	*V. parahaemolyticus*	Nb-based biosensor	Phage–Nb-SH	10^4^ CFU/mL	-	Shrimp	[119]
	*Salmonella Typhimurium*	KNb-DITS	K_0.27_MnO_2_·0. 54 H_2_O@Au@Nb9	Colorimetric mode: 10^4^ CFU/mL Photothermal mode: 10^3^ CFU/mL	-	Juice, honey, and chocolate	[120]
	Aflatoxingenetic fungi	Time-resolved fluorescence immunoassay	PO8-VHH	0.035 μg/mL	0.085~323.56 μg/mL and 0.23~327.55 μg/mL	Blank peanut	[122]

**Table 3:** Recent advances in immunoassay methods for various Pesticide Residues have been based on the use of Nbs.

Principle	Target	Detection Technique	Detection Label	LOD	IC_50_	Linear Range	Sample	Reference
ELISA	Insecticides cyantraniliprole and chlorantraniliprole	C-ELISA	NbC1 and NbC2	0.2 ng mL	1.2 and 1.5 ng/mL	0.4~6.1 ng/mL	Bok choy	[130]
	Dicamba	ic-ELISA	Nb-242	-	0.93 μg/mL	0.11~8.01 μg/mL	Tap water and soil	[131]
	Carbaryl and 1-naphthol	Bic-ELISA	G4S-C-N-VHH	0.8 ng/mL and 0.4 ng/mL	18.8/6.3 ng/mL	2.1~270.9 ng/mL 1.1~112.0 ng/mL	Soil and rice	[132]
Fluorescence immunoassay	Fenitrothion	FIA	VHHjd8-BT	0.03 ng/mL	1.4 ng/mL	0.078~100 ng/mL	Chinese cabbage, lettuce, and tangerine	[133]
	Fenitrothion	FIA	Nb-ALP	5.78 pg/mL	-	0.00001~100 ng/mL	Tap water, river water, apple, chinese cabbage, lettuce, rice, and tomato	[134]
	Quinalphos	PET	Nb-R29W	0.007 μg/mL	0.063 μg/mL	0.015~0.255 μg/mL	Chinese cabbage and cucumber	[142]
LFIA	Parathion	GICA	VHH9	0.15 ng/mL	2.39 ng/mL	0.47~10.58 ng/mL	Chinese cabbage, orange, and cucumber	[136]
	Procymidone	BtNb-ICA	GNP@NbFM5-Bt	0.88 ng/mL	6.04 ng/mL	1.95~18.67 ng/mL	Chives, cucumbers, and tomatoes	[137]
	Paraquat	TRFICA	FM-VHH	0.0090 ng/mL	0.0588 ng/mL	0.0201~0.165 ng/mL	Chinese cabbage, pear, blood, urine, rice, and corn	[138]
Immunosensor	Parathion	Electrochemical immunosensor	VHH9-HRP	2.26 pg/mL	-	0.01~100 ng/mL	Cucumber, orange, and cabbage	[139]
	Fenitrothion	Multicolor immunosensor	VHHjd8ALP	MRVIA: 3.0 ng/mL MRFIA: 1.3 ng/mL	MRVIA: 6.7 ng/mL MRFIA: 6.2 ng/mL	MRVIA: 4.7~11.6 ng/mL MRFIA: 2.6~19.5 ng/mL	Apple, cabbage, and cucumber	[140]
	Fenitrothion	Multicolor immunosensor	VHHjd8ALP	MVIS: 11.2 ng/mL FMVIS: 7.4 ng/mL	MVIS: 70.7 ng/mL FMVIS: 12.1 ng/mL	MVIS: 17.3~197.5 ng/mL FMVIS: 7.7~16.1 ng/mL	Apple, Chinese cabbage, and cucumber	[141]

**Table 4:** Recent advances in immunoassays for various food allergens based on Nbs.

Principle	Target	Detection Technique	Detection Label	LOD	Linear Range	Sample	Reference
ELISA	β-lactoglobulin	cELISA/sELISA	Nb 82	4.55 ng/mL 13.82 ng/mL	39~10,000 ng/mL 29.7~1250 ng/mL	Milk, oatmeal, and candy	[148]
	β-lactoglobulin	sandwich ELISA	Nb 82	0.24 ng/mL	0.01~10 μg/mL	Milk and beverage	[149]
	β-lactoglobulin	sandwich ELISA	HA-Nb	40 pg/mL	3000 pg/mL	Human milk	[150]
	Macadamia protein	sandwich ELISA	Nb 139 H and Nb 68 HA	27.1 ng/mL	0.442~2800 μg/mL	Skimmed milk	[151]
	Ara h 3	sandwich ELISA	Nb P43	53.13 ng/mL	0.2~10.6 μg/mL	Skim milk	[152]
Biosensor	Ara h 1	Nb-μTEI	Nb152	0.86 ng/mL	4.5~55 ng/mL	Milk and chocolate	[153]
	BSA and β-lactoglobulin	“fluorescence–photothermal” immunosensor	Nb82	fluorescence mode: 0.034 ng/mL wavelength mode: 0.075 ng/mL	fluorescence mode: 0.1 ng/mL~0.1 μg/mL wavelength mode: 0.1 ng/mL~0.1 μg/mL	Milk and beverage	[154]
	Ara h 3	colorimetry with ratiometric fluorescence immunoassay	Nb P43	6.61 ng/mL and 9.79 ng/mL	10~1200 ng/mL	Peanut allergy Ara h 3 and fried peanuts	[156]
	Tropomyosin	CM/SERS-LFI	Au^MBA^@AgNPs	0.0026 μg/mL (SERS mode) and 0.0057 μg/mL (colorimetric mode) visual LOD 0.01 μg/mL	0.005~0.5 μg/mL	Bread, cookies, and cheese	[155]
